# Effects of Losartan and Vanillic Acid Co-Administration on Ischemia-Reperfusion-Induced Oxidative Stress in Isolated Rat Heart

**DOI:** 10.5812/ircmj.16664

**Published:** 2014-07-05

**Authors:** Mahin Dianat, Gholam Reza Hamzavi, Mohammad Badavi, Alireza Samarbafzadeh

**Affiliations:** 1 Physiology Research Center, Department of Physiology, Faculty of Medicine, Ahvaz Jundishapur University of Medical Sciences, Ahvaz, IR Iran; 2 Diabetes Research Center, Faculty of Medicine, Ahvaz Jundishapur University of Medical Sciences, Ahvaz, IR Iran; 3 Department of Virology, Faculty of Medicine, Ahvaz Jundishapur University of Medical Sciences, Ahvaz, IR Iran

**Keywords:** Oxidative Stress, Injury, Vanillic Acid, Losartan

## Abstract

**Background:**

Experimental studies have demonstrated that angiotensin II (ANG-II)-induced oxidative stress contributes to the pathogenesis of I/R injury.

**Objectives:**

This study was aimed to investigate the protective effects of co-administration of losartan, as a selective ANG-II type 1 receptor (AT1R) blocker, and vanillic acid (VA), as an antioxidant, in I/R-induced oxidative stress in isolated rat heart.

**Materials and Methods:**

Adult male Wistar rats were randomly divided to sham, control, and five treatment groups (n = 10). Two doses of VA (5 and 10 mg/kg), one dose of losartan (20 mg/kg) alone, and one dose of losartan in combination with either doses of VA were administered orally for 10 days. The hearts were isolated and exposed to 30 minutes ischemia and 60 minutes reperfusion, using Langendorff apparatus. I/R-induced myocardial injury was assessed by estimating the release of lactate dehydrogenase (LDH), creatine phosphokinase (CPK) and myocardial creatine kinase (CK-MB) in coronary effluent at 5, 15, and 60 minutes of reperfusion. The oxidative stress in the hearts was assessed by estimating malondialdehyde (MDA). The effects of treatments on endogenous antioxidant enzymes were assessed by measuring superoxide dismutase (SOD), glutathione peroxidase (GPx) and catalase (CAT).

**Results:**

There was a more significant decrease in the levels of LDH, CPK, CK-MB, and MDA as well as increase in the levels of SOD, CAT and GPx in groups that had received combined treatment in comparison to VA or losartan alone.

**Conclusions:**

It may be concluded that combination of losartan with higher dose of VA decreases ischemic markers and lipid peroxidation and augments endogenous antioxidant and hence, protects myocardium against I/R-induced oxidative stress injuries.

## 1. Background

Accumulating experimental evidence indicates that reperfusion of ischemic myocardium is accompanied by the development of oxidative stress (OS) and the generation of reactive oxygen species (ROS), which play a key role in the etiopathogenesis of reperfusion injury (1-3). The interaction between ROS and cell membrane lipids leads in lipid peroxidation and generation of a cytotoxic product, namely, malondialdehyde (MDA), which in turn lead into membrane disruption, myocardial cell damage, cardiac dysfunction and irreversible tissue injury (4). These events occur with concurrent reduction of certain key endogenous antioxidant compounds such as superoxide dismutase (SOD), glutathione peroxidase (GPx), catalase (CAT) and glutathione (GSH) (5). Moreover, it has been approved that decline in these endogenous antioxidant enzymes activity causes poor functional recovery of myocardium and cardiac tissue injury after ischemia–reperfusion (I/R) (6). Therefore, I/R injuries are the outcome of imbalance between the formation of oxidants (ischemic factors) and the availability of endogenous antioxidants (defensive factors) in the heart. Creatine kinase (CK) is an intramuscular enzyme that has two subunits, namely, M (muscle) and B (brain), which combine to produce isoenzymes CK-MM (muscular), CK-BB (brain), and CK-MB (myocardial). CK and CKMB levels increase with myocardial damage (7). The renin-angiotensin system (RAS) is upregulated during myocardial infarction and I/R injury (8, 9). Angiotensin II (Ang-II), the main effector molecule of the RAS, binds to two major angiotensin receptor subtypes, ie, AT1R and AT2R, to exert its physiological effects; however, several of these interactions increase I/R injury. By stimulating the AT1R, Ang-II causes overexpression of cytosolic proteins involved in the activation of NAD(P)H oxidase, which is a main source of superoxide production (10). One strategy for protecting heart is therefore, reducing Ang-II formation and receptor stimulation with angiotensin-converting enzyme inhibitors (ACEI); however, their effectiveness in I/R is under debate (11). A second strategy is decreasing Ang-II receptor stimulation directly by using selective AT1R or AT2R antagonists. Consequently, these drugs are used widely, particularly in treatment of myocardial infarction, ischemic heart disease (IHD), and hypertension. Due to the effectiveness of these blockers in reducing and/or inhibiting extensive effects of Ang-II, especially to reduce NAD(P)H oxidase activity and hence, superoxide production, we hypothesized that co-administration of an exogenous antioxidant and a selective AT1R blocker could increase defensive factors and reduce ischemic factors, respectively, and would improve management of OS and its consequences. To test this hypothesis, we used VA and Los, as an established antioxidant, radical-scavenging (12) and selective AT1R blocker (13) respectively, and also isolated rat heart was used to induce I/R and subsequently OS.

## 2. Objectives

This study was aimed to investigate the protective effects of co-administration of Losartan and VA in I/R induced oxidative stress in isolated rat heart.

## 3. Materials and Methods

### 3.1. Materials

Losartan and VA were purchased from Sigma-Aldrich Co. (USA) and ketamine HCl (10%) and Xylazine (2%) from Alfasan Co. (the Netherlands). Krebs salts were obtained from Merck Co. (Germany). GPX, MDA, and SOD kits were purchased from Randox Co. (England).

### 3.2. Animals and Treatments

Adult male Wistar rats (body weight, 250-300 g) were randomly divided to seven experimental groups (n = 10) as follow: sham group, control group, V5 group (received 5 mg/kg of VA); V10 group (received 10 mg/kg of VA); Los group (received 20 mg/kg of losartan); V5 + Los group (received 5 mg/kg of VA plus 20 mg/kg of losartan), and V10 + Los group (received 10 mg/kg of VA plus 20 mg/kg of losartan) (8,9). All groups were maintained under the same condition (temperature 22 ± 2℃ and 12-hour dark-light cycle) supplied with food and water ad libitum. Losartan and VA were separately suspended in normal saline and administered to rats via an oral gavage needle for ten days. Control group received normal saline orally for the same duration and sham group received no drug and was not exposed to ischemia. Animals were maintained in the animal house of Jundishapur University of Medical Sciences, Ahvaz, Iran, and treated in accordance with the guidelines of the Animal Care and Use Committee of Laboratory Animals of Ahvaz Jundishapur University of Medical Sciences (No. ajums.REC.1392.91, Date: 4.05.2013).

### 3.3. Preparation of Isolated Heart

Rats were anesthetized with intraperitoneal injection of xylazine (5 mg/kg) and ketamine (50 mg/kg) solution containing heparin (1000 U/kg). After cannulation of trachea, rats were ventilated with room air using a rodent ventilator (UGO BASILE, model: 7025). The thoracic cage was opened; a steel cannula was inserted into the aorta and tightened with a suture. Then, the heart was quickly excised and mounted to a Langendorff perfusion apparatus. The heart perfused at 37 ± 0.1℃ and a constant pressure of 70 mmHg. The perfusion Krebs–Henseleit buffer consisted of NaCl (118 mM), KCl (4.75 mM), CaCl_2 _(1.75 mM), KH_2_PO_4 _(1.18 mM), MgSO_4 _(1.2 mM), NaHCO_3 _(25 mM), and glucose (11.1 mM) in double-distilled water at pH of 7.4 and was equilibrated by 95% O_2_ and 5% CO_2_. For each experiment, fresh perfusion buffer was filtered through a 1.2-µm microfiber filter (GF/C glass filters; Whatman). To allow stabilization of coronary perfusion pressure, all hearts were perfused for 30 minutes before the induction of ischemia, and then subjected to 30 minutes of no-flow global ischemia followed by 60 minutes of reperfusion.

### 3.4. Assessment of Myocardial Injury

I/R-induced myocardial injury was assessed by estimating the release of lactate dehydrogenase (LDH), creatine phosphokinase (CPK), and CK-MB in coronary effluent. To measure these enzymes, perfusate from isolated perfused heart was collected at 5, 15, and 60 minutes after beginning of reperfusion. Spectrophotometric assay was done by standard commercial kits from Sigma-Aldrich (USA) to determine LDH and results were expressed as units per liter (14). CPK and CK-MB levels were measured using immunoinhibition method. In this method, CK activity is measured in the presence of an antibody to CK-M monomer. This antibody completely inhibits the activity of CK-MM and half of the activity of CK-MB, while has no effect on the B subunit activity of CK-MB and CK-BB. Due to the negligible concentrations of CK-BB in the circulation, the remaining activity multiplied by a factor of two represents the activity of the CK-MB isoenzyme (15).

#### 3.4.1. CK-MB Reagent 

The CK-MB reagent (CK reagent, Pointe Scientific Inc., USA) consisted of ADP (2.0 mmol/L), G6PDH (2000 U/L), creatine phosphate (20 mmol/L), NAD (2.0 mmol/L), hexokinase (yeast; 3000 U/L), D-glucose (20 mmol/L), and anti-human CK-M antibody (Mouse; sufficient amount to inhibit up to 2000 U/L of CK-MM at 37℃). In addition, it contained buffers, activators, surfactants and adenylate kinase inhibitors.

#### 3.4.2. Procedure

For each sample and control, 1.0 mL of CK-MB reagent was added into a test tube and warmed for approximately five minutes at 37℃. Then 50 μL of sample was added to its respective tube, mixed well, and incubated for five minutes at 37℃. After five minutes, the absorbance at 340 nm was read and recorded. The increase in absorbance was recorded at 60 second intervals for the next two minutes. The average absorbance per minute (ΔA/Min) was determined and then multiplied by the factor 6752 (3376 × 2) to achieve the results in U/L. Total CK activity was determined according to the manufacturer’s instruction. CK-MB activity was calculated on the basis of the "absorptivity micromolar extinction coefficient" of NADH at 340 nm (0.00622). A unit per liter of CK-MB activity is the amount of enzyme that oxidizes 1 μmol/L of NADH per minute.

### 3.5. Measurement of Antioxidant Enzymes of Heart

At the ending of reperfusion, 500 mg of heart tissue in 5 mL of phosphate buffered saline (PBS; 50 mM at pH of 7.4) was homogenized by a homogenizer (Heidolph Silenterosher M, Germany), and centrifuged at 4000 rpm for ten minutes. The supernatant was assayed for total protein content by total protein kit (Pars Azmun. Co., Iran) (16) along with the activities of SOD, GPx, and CAT. To avoid interassay variation, all analyses were performed, at minimum, in quadruplicate at 25℃ and were assayed on the same day. The coefﬁcients of variation for SOD, GPX, and CAT assays ranged from 2% to 4%.

### 3.6. Myocardial Catalase

CAT levels were measured according to Aebi method (17).

### 3.7. Myocardial Superoxide Dismutase and Glutathione Peroxidase

SOD and GPX levels in the hearts were determined using Randox kits (RandoxLab, UK).

### 3.8. Myocardial Total Antioxidant Capacity 

Total antioxidant capacity (TAC) of the hearts were estimated by Randox kits (RandoxLab, UK).

### 3.9. Myocardial Thiobarbituric Acid Reactive Substances 

Myocardial thiobarbituric acid reactive substances (TBARS), as a marker of lipid peroxidation, was determined using the method described by Ohkawa et al. (18).

### 3.10. Statistical Analysis

Results were expressed as mean ± SEM. Comparisons among groups were performed using one-way ANOVA (for SOD, CAT, GPx, TAC, and MDA) and repeated measurement (for LDH, CPK, and CK-MB), and followed by LSD for multiple comparison tests using SPSS (SPSS Inc., Chicago, IL, USA). P value < 0.05 was considered statistically significant.

## 4. Results

### 4.1. Levels of Lactate Dehydrogenase, Creatine Phosphokinase, and Creatine Kinase-MB in Coronary Effluent

**Figure 1.**
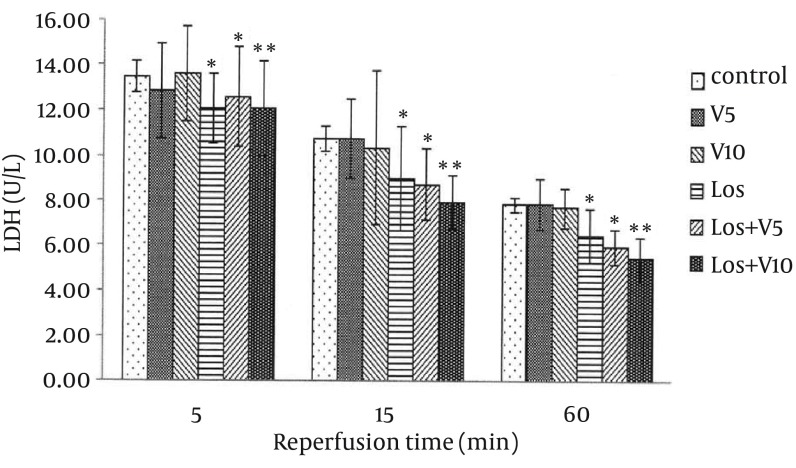
Lactate Dehydrogenase Release in Coronary Effluent of Isolated Rat Hearts Following 30 Minutes Ischemia and 60 Minutes Reperfusion Results are expressed as means ± SEM; n = 10; ^*^, P < 0.05; and ^**^, P < 0.01.

**Figure 2.**
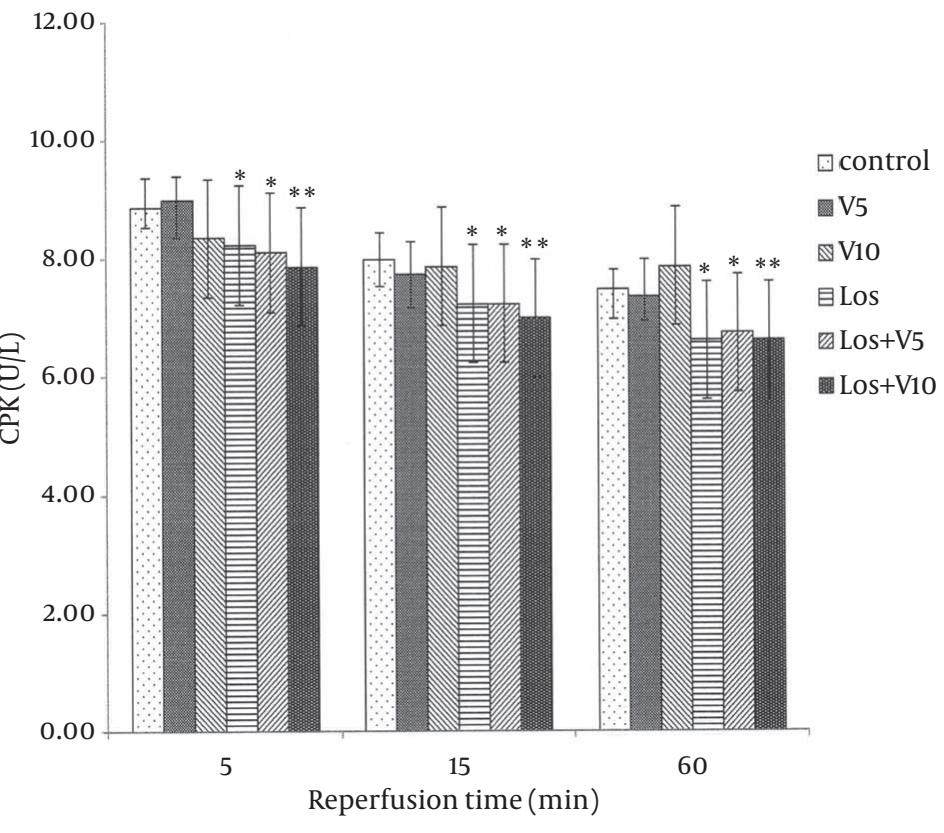
Creatine Phosphokinase Release in Coronary Effluent of Isolated Rat Hearts Following 30 Minutes Ischemia and 60 Minutes Reperfusion Data are expressed as means ± SEM; n = 10; ^*^, P < 0.05; and ^**^, P < 0.01.

**Figure 3.**
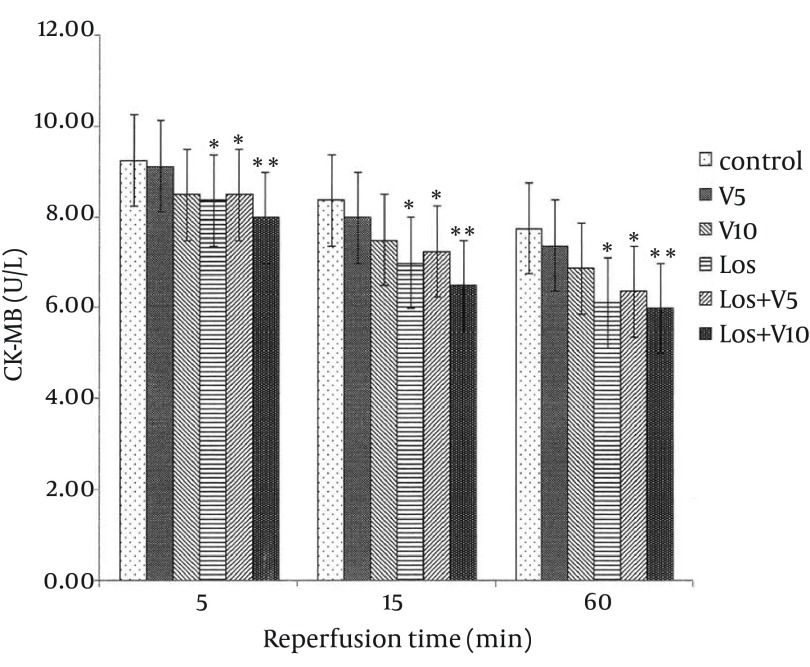
Creatine Kinase-MB Release in Coronary Efﬂuent of Isolated Rat Hearts Following 30 Minutes Ischemia and 60 Minutes Reperfusion Data are expressed as means ± SEM; n = 10; ^*^, P < 0.05; and ^**^, P < 0.01.

As shown in Figures 1, 2, and 3, VA had no significant effects on release of these enzymes in comparison with controls. Pretreatment with losartan caused a significant decline in these enzymes in comparison with controls (P < 0.05). Moreover, combination of losartan with two different doses of VA (Los + V5 and Los + V10) had significant effects in decreasing these ischemic markers in comparison with controls (P < 0.05 and P < 0.01, respectively). The results revealed that co-administration of losartan with higher dose of VA (Los + V10) could decrease these enzymes more significantly than other treated groups.

### 4.2. Level of Lipid Peroxidation

**Figure 4.**
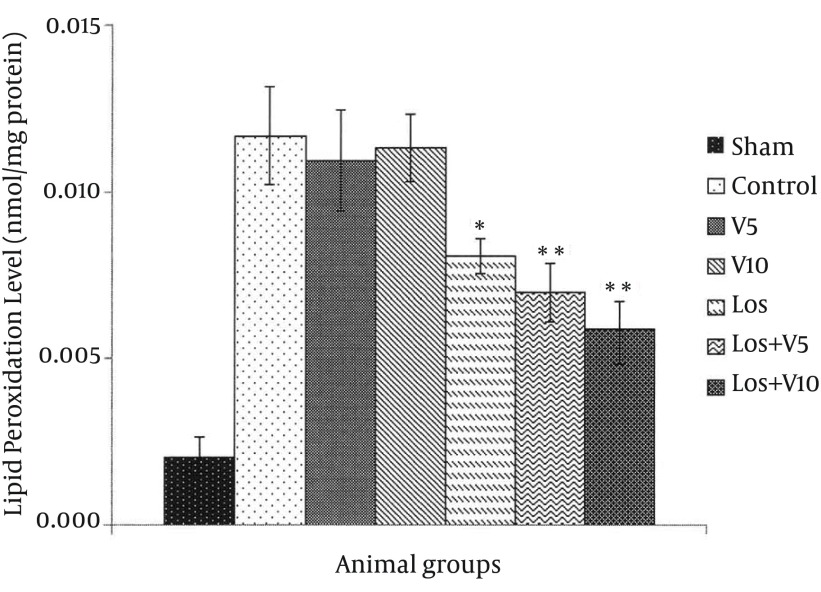
Malondialdehyde Level in Isolated Rat Hearts Following 30 Minutes Ischemia and 60 minutes Reperfusion Data are expressed as means ± SEM; n = 10; *, P < 0.05; and **, P < 0.01.

The levels of MDA in the hearts exposed to global ischemia followed by reperfusion are shown in Figure 4. The results showed that the amounts of MDA in control and all treated groups were higher than the sham group (not exposed to I/R). Although the MDA production in Los group was significantly less than that in control group (P < 0.05), combination of losartan with V5 and V10 resulted to more significant decline in MDA generation in comparison with the control group (P < 0.01).

### 4.3. Myocardial Superoxide Dismutase Level

**Figure 5.**
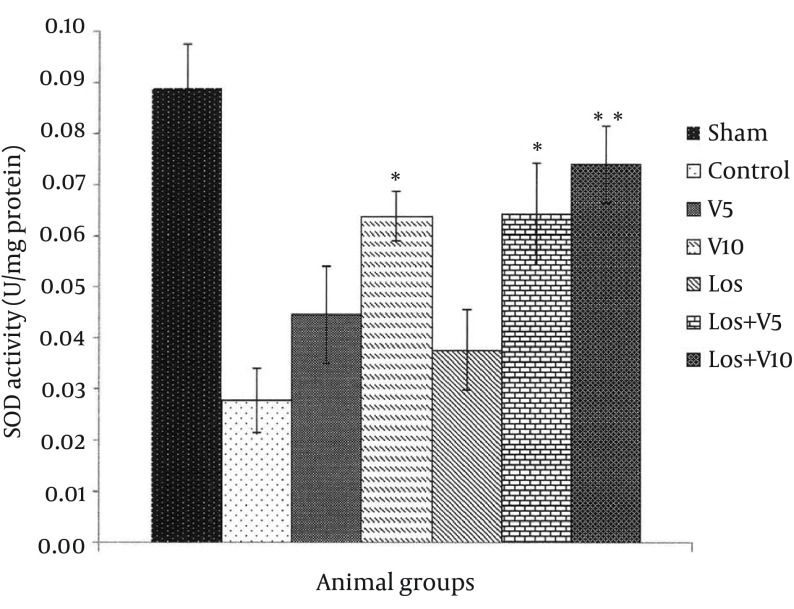
Superoxide Dismutase Level in Isolated Rat Hearts Following 30 Minutes Ischemia and 60 Minutes Reperfusion Data are expressed as means ± SEM; n = 10; *, P < 0.05; and **, P < 0.01.

SOD changes in all groups are shown in Figure 5. As compared with the sham group, the amounts of SOD in the hearts of control and treated groups decreased after exposure to I/R and consequent OS. Based on these results, V5 and Los groups had no significant effects in the SOD activity in comparison with controls; however, in other treated groups including V10 (P < 0.05), Los + V5 (P < 0.05), and Los + V10 (P < 0.01), the SOD level increased significantly in comparison with control group.

### 4.4. Myocardial Catalase Level

**Figure 6.**
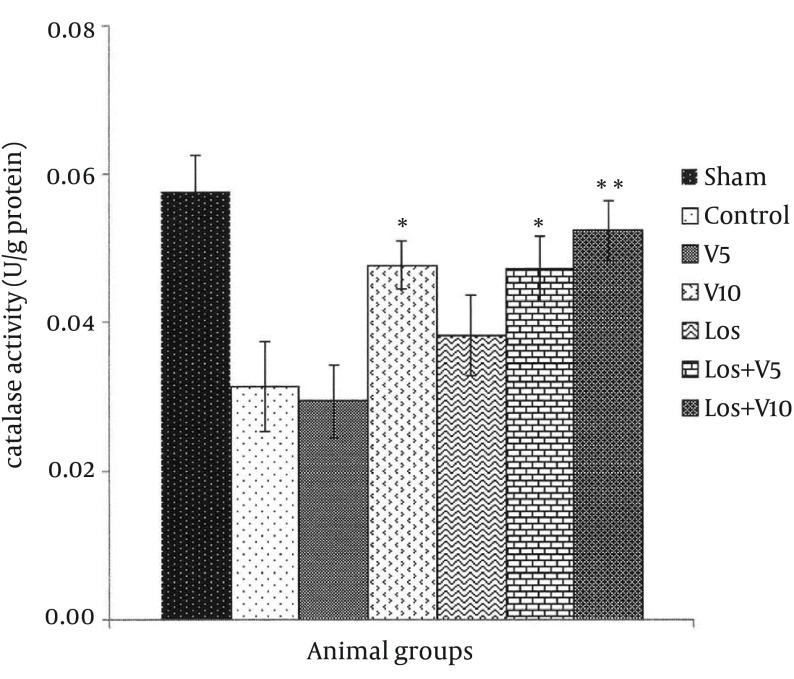
Catalase Level in Isolated Rat Hearts Following 30 Minutes Ischemia and 60 Minutes Reperfusion Data are expressed as means ± SEM; n = 10; *, P < 0.05; and **, P < 0.01.

As shown in Figure 6, CAT levels reduced in all groups exposed to I/R in comparison with sham; however, the levels of CAT in the hearts of V10, Los + V5, and Los + V10 groups enhanced significantly in comparison with control group (P < 0.05, P < 0.05, and P < 0.01, respectively). There were no significant changes in the CAT levels in V5 and Los groups.

### 4.5. Myocardial Glutathione Peroxidase Level

**Figure 7.**
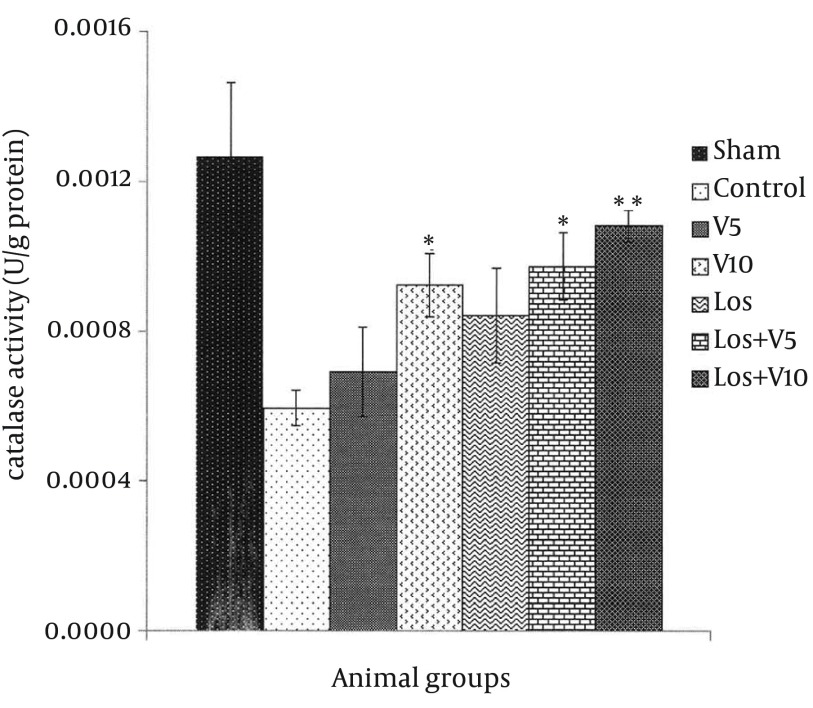
Glutathione Peroxidase Level in Isolated Rat Hearts Following 30 Minutes ischemia and 60 Minutes Reperfusion Data are expressed as means ± SEM; n = 10; *, P < 0.05; and **, P < 0.01.

The same as other antioxidant enzymes levels presented above, the amounts of GPx in the hearts exposed to I/R decreased in comparison with the sham group (Figure 7). According to these results and in comparison with control group, pretreatment with V10, Los + V5, and Los + V10 significantly increased the levels of GPx, (P < 0.05, P < 0.05, and P < 0.01, respectively). The levels of GPx in the V5 and Los groups did not alter markedly.

### 4.6. Myocardial Total Antioxidant Capacity

**Figure 8.**
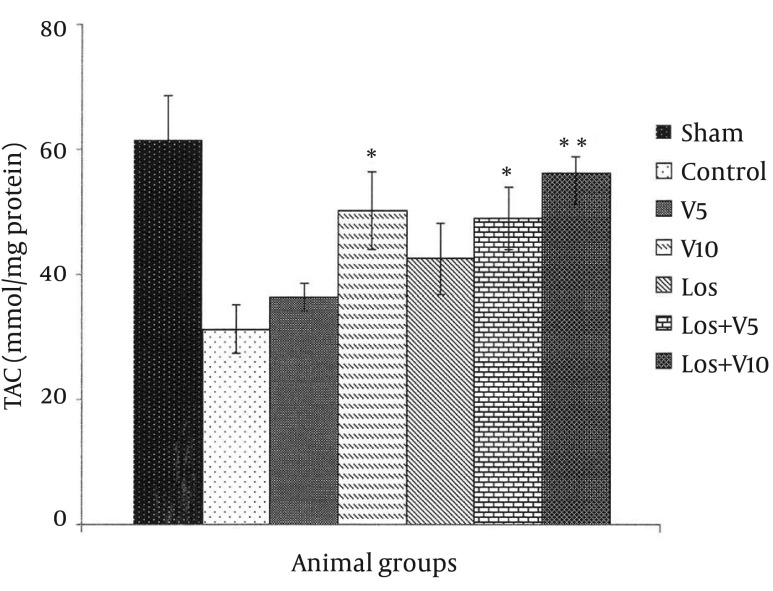
Total Antioxidant Capacity Level in Isolated Rat Hearts Following 30 Minutes ischemia and 60 Minutes Reperfusion Data are expressed as means ± SEM; n = 10; *, P < 0.05; and **, P < 0.01.

TAC decreased in all groups after I/R, in comparison with sham group (Figure 8). Our treatments increased this capacity in comparison with control group; however, the significant alternations of TAC were observed only in V10 (P < 0.05), Los + V5 (P < 0.05), and Los + V10 (P < 0.01) groups in comparison to control; V5 and Los groups did not show marked changes in TAC in comparison with the controls.

## 5. Discussion

We examined the combination of an AT1R blocker with an exogenous antioxidant as a strategy for better management of myocardial injury due to I/R-induced OS. Our results were consistent with previous reports that indicated the protective effects of VA on cardiac troponins, lipid peroxidation, antioxidant system, electrocardiogram, expressions of interleukin 1 beta, interleukin 6, infarct size, and biochemical parameters in the heart of isoproterenol-induced cardiotoxic rats (19, 20). Our results demonstrated the effectiveness of VA on lipid peroxidation, indicated by a decrease in MDA, and enhancement of endogenous antioxidant enzymes, indicated by increased SOD, CAT, GPx, and TAC in the rat hearts exposed to I/R. 

The extent of CK-MB elevation strongly correlates with infarct size; moreover, LDH raises in myocardial infarction after reperfusion, which may be due to sustained ischemic injury (7). In the present study, 30 minutes ischemia followed by 60 min reperfusion produced myocardial injury as shown by elevated levels of CPK, CK-MB, and LDH in the coronary effluent, which was consistent with the earlier reports (21, 22). 

Lipid peroxidation refers to oxidative reduction of lipids. In this process, free radicals steal electron from the lipids in cell membrane and result in cell damage (7). TBARS are low-molecular end products, the major component of which is MDA, that are made during the decomposition of lipid peroxidation product. SOD, CAT, and GPx are the main endogenous antioxidants that play a critical role in the cellular defense against OS. In the present study, losartan effectively decreased LDH, CPK, and CK-MB, which has been demonstrated previously (23); however, combination of losartan with 10 mg/kg of VA more significantly lowered these enzymes, which means more protective effect against myocardial injury. Moreover, co-administration of losartan with both doses of VA reduced MDA more than losartan alone. Taken these results together with more significant effects of this combination on enhancing the levels of antioxidant enzymes in comparison with any of them alone, showed that losartan and VA synergistically potentiated their protective effects against OS and hence, reduced myocardial damage. It has been shown that selective inhibition of AT1R reduces OS not only by decreasing NAD(P)H oxidase activity, but also by selectively increasing SOD expression and its activity, primarily in the perivascular area and media of the intramyocardial arteries and cardiac myocytes (24). In Addition, treatment with SOD mimetic has been shown to reduce OS, improve vascular function and structure, and prevent the progression of hypertension in SHR (spontaneously hypertensive rat) via altering the activation of NAD(P)H oxidase and Cu/Zn SOD (25); however, the SOD isoforms, GPX, and CAT reside within the cells that finally lead to elimination of free radicals by generation of water and oxygen (26). There is also evidence that various plants and plant extracts can stimulate the synthesis of cellular antioxidants (3, 27, 28). 

In this study, decreasing of MDA and ischemic markers showed the preventive effects of losartan on OS that can be due to alternation of NADPH oxidase activity and ROS production. As current study showed, combination of losartan and VA resulted in more decrease in indicators of OS. The possible explanation for this synergistic effects is the augmented inhibition of NADPH oxidase activity due to the antioxidant and radical-scavenging properties of VA; moreover, VA ability to increase SOD, CAT, and GPx contributes not only to the elimination of free radicals, but also to the inhibition of NADPH oxidase activity by augmenting SOD. Consistent with the beneficial effects of AT1R blocker in combination with other compounds, has been shown that combination of olmesartan and pravastatin exerted positive vascular effects in salt-sensitive hypertension (29), co-administration of olmesartan and 17-β-estradiol synergistically attenuated atherosclerosis at least partly via inhibition of OS (10), and concomitant administration of fluvastatin and valsartan blunted OS, inflammation and atherosclerosis (26). 

In conclusion, we demonstrated that the combination of losartan and VA synergistically reduced OS, which was shown as MDA reduction, declined myocardial injury shown as decrease in ischemic markers, and augmented endogenous antioxidant enzymes including SOD, CAT, and GPx.

## References

[1] Petrosillo G, Ruggiero FM, Di Venosa N, Paradies G (2003). Decreased complex III activity in mitochondria isolated from rat heart subjected to ischemia and reperfusion: role of reactive oxygen species and cardiolipin.. FASEB J..

[2] Zhu X, Liu B, Zhou S, Chen YR, Deng Y, Zweier JL (2007). Ischemic preconditioning prevents in vivo hyperoxygenation in postischemic myocardium with preservation of mitochondrial oxygen consumption.. Am J Physiol Heart Circ Physiol..

[3] Gauthaman KK, Saleem MT, Thanislas PT, Prabhu VV, Krishnamoorthy KK, Devaraj NS (2006). Cardioprotective effect of the Hibiscus rosa sinensis flowers in an oxidative stress model of myocardial ischemic reperfusion injury in rat.. BMC Complement Altern Med..

[4] Kalaycioglu S, Sinci V, Imren Y, Oz E (1999). Metoprolol prevents ischemia-reperfusion injury by reducing lipid peroxidation.. Jpn Circ J..

[5] Liu P, Xu B, Cavalieri TA, Hock CE (2004). Attenuation of antioxidative capacity enhances reperfusion injury in aged rat myocardium after MI/R.. Am J Physiol Heart Circ Physiol..

[6] Venardos K, Harrison G, Headrick J, Perkins A (2004). Auranofin increases apoptosis and ischaemia-reperfusion injury in the rat isolated heart.. Clin Exp Pharmacol Physiol..

[7] Singh G, Rohilla A, Singh M, Balakumar P (2009). Possible role of JAK-2 in attenuated cardioprotective effect of ischemic preconditioning in hyperhomocysteinemic rat hearts.. Yakugaku Zasshi..

[8] Horiuchi M, Akishita M, Dzau VJ (1999). Recent progress in angiotensin II type 2 receptor research in the cardiovascular system.. Hypertension..

[9] Flynn JD, Akers WS (2003). Effects of the angiotensin II subtype 1 receptor antagonist losartan on functional recovery of isolated rat hearts undergoing global myocardial ischemia-reperfusion.. Pharmacotherapy..

[10] Tsuda M, Iwai M, Li JM, Li HS, Min LJ, Ide A (2005). Inhibitory effects of AT1 receptor blocker, olmesartan, and estrogen on atherosclerosis via anti-oxidative stress.. Hypertension..

[11] Przyklenk K, Kloner RA (1993). "Cardioprotection" by ACE-inhibitors in acute myocardial ischemia and infarction?. Basic Res Cardiol..

[12] Tai A, Sawano T, Ito H (2012). Antioxidative properties of vanillic acid esters in multiple antioxidant assays.. Biosci Biotechnol Biochem..

[13] Yao HW, Zhu JP, Zhao MH, Lu Y (2006). Losartan attenuates bleomycin-induced pulmonary fibrosis in rats.. Respiration..

[14] Zhou HZ, Swanson RA, Simonis U, Ma X, Cecchini G, Gray MO (2006). Poly(ADP-ribose) polymerase-1 hyperactivation and impairment of mitochondrial respiratory chain complex I function in reperfused mouse hearts.. Am J Physiol Heart Circ Physiol..

[15] Babinska M, Holecki M, Prochaczek F, Owczarek A, Kokocinska D, Chudek J (2012). Is plasma urotensin II concentration an indicator of myocardial damage in patients with acute coronary syndrome?. Arch Med Sci..

[16] Parlakpinar H, Olmez E, Acet A, Ozturk F, Tasdemir S, Ates B (2009). Beneficial effects of apricot-feeding on myocardial ischemia-reperfusion injury in rats.. Food Chem Toxicol..

[17] Aebi H (1984). Catalase in vitro.. Methods Enzymol..

[18] Ohkawa H, Ohishi N, Yagi K (1979). Assay for lipid peroxides in animal tissues by thiobarbituric acid reaction.. Anal Biochem..

[19] Prince PS, Dhanasekar K, Rajakumar S (2011). Preventive effects of vanillic acid on lipids, bax, bcl-2 and myocardial infarct size on isoproterenol-induced myocardial infarcted rats: a biochemical and in vitro study.. Cardiovasc Toxicol..

[20] Stanely Mainzen Prince P, Rajakumar S, Dhanasekar K (2011). Protective effects of vanillic acid on electrocardiogram, lipid peroxidation, antioxidants, proinflammatory markers and histopathology in isoproterenol induced cardiotoxic rats.. Eur J Pharmacol..

[21] Asdaq SM, Inamdar MN (2009). Pharmacodynamic interaction of garlic with hydrochlorothiazide in rats.. Indian J Physiol Pharmacol..

[22] Asdaq SM, Inamdar MN, Asad M (2010). Pharmacodynamic interaction of garlic with propranolol in ischemia-reperfusion induced myocardial damage.. Pak J Pharm Sci..

[23] Paz Y, Gurevitch J, Frolkis I, Matsa M, Kramer A, Locker C (1998). Effects of an angiotensin II antagonist on ischemic and nonischemic isolated rat hearts.. Ann Thorac Surg..

[24] Tanaka M, Umemoto S, Kawahara S, Kubo M, Itoh S, Umeji K (2005). Angiotensin II type 1 receptor antagonist and angiotensin-converting enzyme inhibitor altered the activation of Cu/Zn-containing superoxide dismutase in the heart of stroke-prone spontaneously hypertensive rats.. Hypertens Res..

[25] Park JB, Touyz RM, Chen X, Schiffrin EL (2002). Chronic treatment with a superoxide dismutase mimetic prevents vascular remodeling and progression of hypertension in salt-loaded stroke-prone spontaneously hypertensive rats.. Am J Hypertens..

[26] Li Z, Iwai M, Wu L, Liu HW, Chen R, Jinno T (2004). Fluvastatin enhances the inhibitory effects of a selective AT1 receptor blocker, valsartan, on atherosclerosis.. Hypertension..

[27] Karthikeyan K, Bai BR, Gauthaman K, Sathish KS, Devaraj SN (2003). Cardioprotective effect of the alcoholic extract of Terminalia arjuna bark in an in vivo model of myocardial ischemic reperfusion injury.. Life Sci..

[28] Gauthaman K, Banerjee SK, Dinda AK, Ghosh CC, Maulik SK (2005). Terminalia arjuna (Roxb.) protects rabbit heart against ischemic-reperfusion injury: role of antioxidant enzymes and heat shock protein.. J Ethnopharmacol..

[29] Yamamoto E, Yamashita T, Tanaka T, Kataoka K, Tokutomi Y, Lai ZF (2007). Pravastatin enhances beneficial effects of olmesartan on vascular injury of salt-sensitive hypertensive rats, via pleiotropic effects.. Arterioscler Thromb Vasc Biol..

